# Under or Absent Reporting of Light Stimuli in Testing of Anxiety-Like Behaviors in Rodents: The Need for Standardization

**DOI:** 10.3389/fnmol.2022.912146

**Published:** 2022-08-17

**Authors:** Lorenz S. Neuwirth, Michael T. Verrengia, Zachary I. Harikinish-Murrary, Jessica E. Orens, Oscar E. Lopez

**Affiliations:** ^1^Department of Psychology, SUNY Old Westbury, Old Westbury, NY, United States; ^2^SUNY Neuroscience Research Institute, SUNY Old Westbury, Old Westbury, NY, United States

**Keywords:** light/dark test, open field, elevated plus maze, three chamber social interaction test, Lux/lighting, behavioral neuroscience tests, rodent pre-clinical screening/testing, ethologically motivated controls

## Abstract

Behavioral neuroscience tests such as the Light/Dark Test, the Open Field Test, the Elevated Plus Maze Test, and the Three Chamber Social Interaction Test have become both essential and widely used behavioral tests for transgenic and pre-clinical models for drug screening and testing. However, as fast as the field has evolved and the contemporaneous involvement of technology, little assessment of the literature has been done to ensure that these behavioral neuroscience tests that are crucial to pre-clinical testing have well-controlled ethological motivation by the use of lighting (i.e., Lux). In the present review paper, *N* = 420 manuscripts were examined from 2015 to 2019 as a sample set (i.e., *n* = ~20–22 publications per year) and it was found that only a meager *n* = 50 publications (i.e., 11.9% of the publications sampled) met the criteria for proper anxiogenic and anxiolytic Lux reported. These findings illustrate a serious concern that behavioral neuroscience papers are not being vetted properly at the journal review level and are being released into the literature and public domain making it difficult to assess the quality of the science being reported. This creates a real need for standardizing the use of Lux in all publications on behavioral neuroscience techniques within the field to ensure that contributions are meaningful, avoid unnecessary duplication, and ultimately would serve to create a more efficient process within the pre-clinical screening/testing for drugs that serve as anxiolytic compounds that would prove more useful than what prior decades of work have produced. It is suggested that improving the standardization of the use and reporting of Lux in behavioral neuroscience tests and the standardization of peer-review processes overseeing the proper documentation of these methodological approaches in manuscripts could serve to advance pre-clinical testing for effective anxiolytic drugs. This report serves to highlight this concern and proposes strategies to proactively remedy them as the field moves forward for decades to come.

## Introduction

Over the last decade, there has been a rapid advancement of a more integrative and interdisciplinary neuroscience that has sought to explore structure-function relationships either through brain mapping, (neuro) imaging, establishing and refining the connectome, and/or using cross-species comparisons to better understand the uniqueness of the human brain that orchestrate behavioral outputs (Koch et al., 2010; Goulas et al., 2014; Miranda-Dominquez et al., 2014; Wang et al., 2014; Snyder and Bauer, 2019). Despite these exceptional advancements, prior to this recent boom in the big-technology and big-data revolution in the field of neuroscience, there remained a series of challenges faced by behavioral neuroscientists in trying to address longstanding issues in: relating behavioral observations back to molecular biological targets and exercising caution that molecular data cannot replace the unique adaptive behavioral processes of organisms in response to changing environments (Lederhendler and Schulkin, 2000), mining large datasets (Akil et al., 2011) and in particular bridging data between psychology, ethology, and neuroscience (Marshall, 2009; Gomez-Marin et al., 2014), with long-term goals in understanding the subtleties of social behaviors (Adolphs, 2010; Insel, 2010) that can be complex in nature (Cacioppo and Decety, 2011), and to remain grounded in our understanding of reinforcement learning, conditioning, and its neural correlates (Maia, 2009). What is more concerning is that these challenges remain decades later with little advancement and standardization in their use within the field to address such a critical problem.

This situation may be partially explained by many non-traditionally trained behaviorists engaging in basic behavioral neuroscience tests in an effort to characterize and/or determine traits of specific locomotor activities (i.e., through the use of the Open Field) and anxiety-like behaviors (i.e., through the use of the Elevated Plus Maze, Zero Maze, Light/Dark Box or Light/Dark Test, and the Three Chamber Social Interaction Test). This situation has resulted in an unfortunate reduction in the quality of behavioral neuroscience research (Bespalov and Steckler, 2018) and efforts by other groups to try to make behavioral tests they design more accessible through open-source applications are noteworthy (White et al., 2019). Notably, without ensuring both generalizability and reliability training through open-source efforts to standardize the field, one must seriously question the behavioral neuroscience datasets they review as their interpretations can be quite variable. There seems to be a tendency that as advancements in (bio) technology are made, it inadvertently creates a widening gap between re-establishing important links of behavioral bioassays in neuroscience (Brown and Bolivar, 2018). Thus, researchers in the field that employ behavioral tests to better describe neurobiological phenomena associated with a broad range of animal model systems and associated ecological contexts to diversify the translations of their work (Mathuru et al., 2020) should undergo more training in traditional behaviorism (Thompson, 1994; Moore, 2011) to prevent creating barriers in integrating behavioral and neural datasets (Thompson, 1994; Moore, 2002; Carobrez and Bertoglio, 2005; Ortu and Vaidya, 2016).

One of the basic tenets of the Open Field Test, the Elevated Plus Maze, the Zero Maze, the Light/Dark Test, or the Light/Dark Box, is the use of an aversive bright light to serve as an establishing operation to induce anxiogenic behaviors, whereas, in contrast, the Three Chamber Social Interaction Test uses dim lighting to increase socialization to induce anxiolytic behaviors. These ethologically derived concepts surrounding the intentional utilization of light as the motivational stimuli serve as the foundational basis by which all interpreted anxiety-like behaviors in rodents are reliably confirmed through pre-clinical behavioral neuroscience testing procedures. Thus, the rodent’s sensitivity to lighting (i.e., changes in Lux) can serve to set the floor and ceiling thresholds for anxiogenic responses in both typical wild type, transgenic, and/or other mutant rodent models engineered to exhibit face and construct valid social and other associated anxiety-like behavioral traits. However, despite cases where revisiting standards for testing anxiety-like behaviors for the Elevated Plus Maze over a 20-year period have been reported in the literature to increase future face, construct, and predictive validity within an ethological context (Ortu and Vaidya, 2016), it unfortunately, failed to include lighting controls in its 2-decade review of the experimentation. These observations led to the following question: How many publications for these behavioral neuroscience tests actually report the Lux values as their ethological stimulus controls for inducing anxiogenic and/or anxiolytic responses? This question is important to consider, especially if the behavioral neuroscience field continues to produce large amounts of publications without the standardization of Lux through clearly defined controlled lighting as the main experimental control to be the anxiogenic/anxiolytic stimulus to motivate rodents. Furthermore, when such standardization is established, it, in turn, increases the integrity of the science, the internal and external validity, and translational meaning of the work that has and continues to be published and the work forthcoming. Thus, the present study sought to determine what proportion of research studies actually report the lighting/lux values in their reports in an effort to revisit the standards for behavioral neuroscience testing of anxiety-like behaviors in rodents.

## Method

### Database Keyword Search

In order to determine the frequency of publications that used the Open Field Test, the Elevated Plus Maze, the Zero Maze, the Light/Dark Box, or the Light/Dark Test, and the Three Chamber Social Interaction Test, a keyword search for these exact terms were done using Elsevier’s Science Direct for the years 2009–2019 covering a decade of research published through this outlet. The Elsevier’s Science Direct Article Database was used as it had to most journal subscriptions (i.e., 4,620 journals) to cover a broad range to obtain a representative sample of the behavioral neuroscience tests understudy. The years were truncated not to go beyond 2019 to avoid any publication data being skewed due to the coronavirus (COVID-19). However, some papers in 2019 at the time of the search were either in press/forthcoming or published online ahead of print and their actual publication dates occurred in 2020. The total number of the articles returned that met the inclusion criteria were then used to evaluate the trends of the use of each test in the behavioral neuroscience field over the last decade.

### Refined Search and Inclusion/Exclusionary Criteria

**Figure 1:**
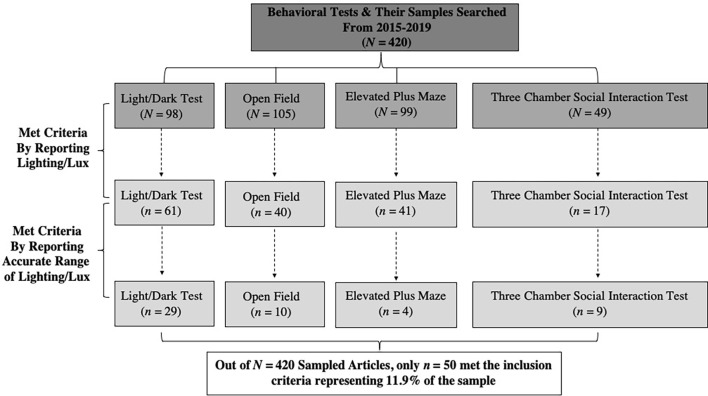
This figure illustrates the flow chart of how the 420 behavioral articles that were searched for to generate an equal representative sample (*n* = 105 articles of each behavioral test) comprising the Light/Dark Test, Open Field Test, Elevated Plus Maze Test, and the Three Chamber Social Interaction Test were sampled from 2015 to 2019 (*n* = 20–22 per year; **upper dark gray rectangles**). Of the *N* = 420 sampled, only *n* = 351 met the criteria for animal behavioral test relevance. The publications were then examined for meeting the inclusion criteria for reporting a lighting/Lux value for ethological controls for either anxiogenic or anxiolytic responses (**middle light gray rectangles**). Next, the refined number of articles were then examined for meeting the inclusion criteria for reporting accurate ranges of lighting/Lux for ethologically relevant stimuli motivation purposes that align with the test’s purpose in the field of behavioral neuroscience (**lower light gray rectangles**). Through the criteria used, 11.9% of the sampled articles across the behavioral tests were published using appropriate ethological motivational principles (**white lowest rectangle**) and only *n* = 50 met the full inclusion criteria, with the Light/Dark Test having better reporting (62.25%), followed by the Open Field (30.09%), then the Elevated Plus Maze (41.41%), and finally the Three Chamber Social Interaction Test with the worst (34.69%) of the respective samples.

Next, for each of the types of behavioral tests of interest (i.e., Light/Dark Box, the Open Field, the Elevated Plus Maze, and the Three Chamber Social Interaction Test) from 2015 to 2019, covering the latter half of the decade (i.e., since there was a consistent upward trend in these latter years). Using this time-period, the first 22–23 publications from each year (i.e., 105 publications per behavioral test that were sampled; totaling *N* = 420 publications of which only *n* = 351 met the criteria (Kochenborger et al., 2014; Allah Yar et al., 2015; Amos-Kroohs et al., 2015; Banaskowski et al., 2015; Bentea et al., 2015; Bernard et al., 2015; Bertolus et al., 2015; Brown et al., 2015; Bruining et al., 2015; Casarrubea et al., 2015, 2016; Chao et al., 2015; Colla et al., 2015; Cox et al., 2015; Daher and Mattioli, 2015; Dutra-Tavares et al., 2015; Finlay et al., 2015; Fowler and Muma, 2015; Gamberini et al., 2015; Goes et al., 2015; Gray and Hughes, 2015; Haleem et al., 2015; Hill et al., 2015; Horii and Kawaguchi, 2015; Iqbal et al., 2015; Kalouda and Pitsikas, 2015; Kawasaki et al., 2015, 2017; Ketcha Wanda et al., 2015; Kigar et al., 2015; Kumar et al., 2015; Langley et al., 2015; Lawther et al., 2015; Lecorps and Féron, 2015; Lee J. et al., 2015; Lee K. M. et al., 2015; Listowska et al., 2015; Liu et al., 2015; Livingston-Thomas et al., 2015; Mascarenhas et al., 2015; Mcneilly et al., 2015; Nakamura A. et al., 2015; Nakamura K. et al., 2015; Noguerón-Merino et al., 2015; Nunes et al., 2015; Pereda et al., 2015; Quines et al., 2015; Rafati et al., 2015; Reilly et al., 2015; Reimer et al., 2015; Rilett et al., 2015; Saito and Brandão, 2015; Saitoh et al., 2015; Sauce et al., 2015; Schroeder et al., 2015; Skupio et al., 2015; Słupski and Rutkowska, 2015; Štefánik et al., 2015; Telonis and Margarity, 2015; Thompson et al., 2015; Tothova et al., 2015; Washington et al., 2015; Wensheng et al., 2015; Xu et al., 2015, 2019; Yang et al., 2015; Yoshimi et al., 2015; Yu et al., 2015; Zagorácz et al., 2015; Zhan, 2015; Zhang C. et al., 2015; Zhang H. et al., 2015; Zhang M. et al., 2015; Zhang W. et al., 2015; Zhou et al., 2015; Zoubovsky et al., 2015; Acevedo et al., 2016; Bashiri et al., 2016; Biagioni et al., 2016; Buffington et al., 2016; Christensen et al., 2016; Cipriano et al., 2016; Dagan et al., 2016; Daniel and Hughes, 2016; de La Tremblaye et al., 2016; Dhediya et al., 2016; Diaz et al., 2016; Dos Anjos et al., 2016; Duarte et al., 2016; Estork et al., 2016; Farajdokht et al., 2016; Fedotova et al., 2016; Fernandez et al., 2016; Ferri et al., 2016; Figueiredo et al., 2016; Girard et al., 2016; Gomes et al., 2016; Gomez et al., 2016; Hegde et al., 2016; Hicks K. et al., 2016; Hicks J. A. et al., 2016; Horsley et al., 2016; Hughes and Hancock, 2016; Kang et al., 2016; Kerr et al., 2016; Komaki et al., 2016; Kratsman et al., 2016; Kumar and Sharma, 2016a, b; Labots et al., 2016; Lamontagne et al., 2016; Lawson et al., 2016; Lecorps et al., 2016; Lee and Green, 2016; Lee et al., 2016, 2018; Li J. et al., 2016; Li K. et al., 2016; Martins-Júnior et al., 2016; Matthews et al., 2016; Miller et al., 2016; Miranda-Morales and Pautassi, 2016; Mlyniec et al., 2016; Näslund et al., 2016; O’Connor et al., 2016; Onaolapo et al., 2016; Palotai and Telegdy, 2016; Psyrdellis et al., 2016a, b; Rico et al., 2016; Rodrigues Tavares et al., 2016; Rogers et al., 2017; Rojas et al., 2016; Ryan et al., 2016; Sakurai et al., 2016; Salari et al., 2016; Saré et al., 2016; Schambra et al., 2016; Scheinert et al., 2016; Serafim et al., 2016; Socala and Wlaz, 2016; Stohn et al., 2016; Torres et al., 2016; Tran and Keele, 2016; Verpeut et al., 2016; Vogt et al., 2016; Wang et al., 2016a, b, 2018b; Wscieklica et al., 2016; Yau et al., 2016; Yeung et al., 2016; Zhang et al., 2016; Abdellatif et al., 2017; Akbar et al., 2017; Alkhlaif et al., 2017; Bagosi et al., 2017a, b; Bahi, 2017a, b; Bartolomé et al., 2017; Bassi et al., 2017; Benoit et al., 2017; Blankenship et al., 2017; Borbely et al., 2017; Burke and Trang, 2017; Cai et al., 2017; Cao et al., 2017; Casarrubea et al., 2017; Chandra Sekhar et al., 2017; Demir Özkay et al., 2017; Djordjevic et al., 2017; Domonkos et al., 2017; Donatti et al., 2017; Ebihara et al., 2017; Fernandes et al., 2017a, b; Garcia et al., 2017; Gatica et al., 2017; Gillette et al., 2017; Hansen et al., 2017; Henbid et al., 2017; Hsieh et al., 2017; Jiménez-Ferrer et al., 2017; Karkaba et al., 2017; Kawabe, 2017; Kędzierska et al., 2017; Khalil and Fendt, 2017; Kuniishi et al., 2017; Kyriakou et al., 2017; López-Cruz et al., 2017; Lee et al., 2017; Leković et al., 2017; Machado et al., 2017; Makinson et al., 2017; Malikowska and Sałat, 2017; Mazur et al., 2017; Mihara et al., 2017; Narasingam et al., 2017; Orfanidou et al., 2017; Provenzano et al., 2017; Rangel-Barajas et al., 2017; Reinhart et al., 2017; Salari and Amani, 2017; Sanguedo et al., 2017; Sanna et al., 2017; Santangelo et al., 2017; Santos et al., 2017; Scheich et al., 2017; Schindler et al., 2017; Shafia et al., 2017; Sirohi et al., 2017; Speight et al., 2017; Sprowles et al., 2017; Stoppel and Anderson, 2017; Taherichadorneshin et al., 2017; Toma et al., 2017; Uemura et al., 2017; van Den Boom et al., 2017; Vázquez-León et al., 2017, 2018; Wei et al., 2017; Wen et al., 2017; Wu et al., 2017; Yamamoto et al., 2017; Yeshurun et al., 2017; Zilkha et al., 2017; Zimcikova et al., 2017; Alves et al., 2018; Amodeo et al., 2018; Arakawa, 2018; Batinić et al., 2018; Bausch et al., 2018; Benekareddy et al., 2018; Bialuk et al., 2018; Blume et al., 2018; Bodden et al., 2018; Bonuti and Morato, 2018; Borland et al., 2018; Boyette-Davis et al., 2018; Cazuza et al., 2018; Crestani et al., 2018; Cui et al., 2018; Dastamooz et al., 2018; Donaire et al., 2018; Dong et al., 2018; Evans et al., 2018; Ferreira de Araújo et al., 2018; Funck et al., 2018; Garcia-Gutierrez et al., 2018; Goñi-Balentziaga et al., 2018; He et al., 2018; Heinla et al., 2018; Hirano et al., 2018; Holman et al., 2018; Holubová et al., 2018; Hughes and Hamilton, 2018; Jacobskind et al., 2018; Keenan et al., 2018; Khalil et al., 2018; Kosari-Nasab et al., 2018; Leung et al., 2018; López Rivilli et al., 2018; Macedo et al., 2018; Mahmoudi et al., 2018; Melo et al., 2018; Melo-Thomas et al., 2018; Morley-Fletcher et al., 2018; Morud et al., 2018; Namvarpour et al., 2018; Nie et al., 2018; Noworyta-Sokołowska et al., 2018; Okamoto et al., 2018; Perea-Rodriguez et al., 2018; Purvis et al., 2018; Rauhut and Curran-Rauhut, 2018; Robinson et al., 2018; Rojas-Carvajal et al., 2018; Roohi-Azizi et al., 2018; Saitoh et al., 2018; Screven and Dent, 2018; Sheth et al., 2018; Shimizu et al., 2018; Smith et al., 2018; Sobolewski et al., 2018; Sorregotti et al., 2018; Sparling et al., 2018; Struntz and Siegel, 2018; Subramaniam et al., 2018; Tarland and Brosda, 2018; Tavares et al., 2018; Upadhyay et al., 2018; Van Camp et al., 2018; Varghese et al., 2018; Walia et al., 2018, 2019a, b; Wang S. et al., 2018; Wang et al., 2018a, b; Wille-Bille et al., 2018; Zahra et al., 2018; Zhang and Yao, 2018; Zhou et al., 2018; Al-Harrasi et al., 2019; Arnold et al., 2019; Atigari et al., 2019; Bahi and Dreyer, 2019; Basaure et al., 2019; Borrow et al., 2019; Burns et al., 2019; Caliskan et al., 2019; Dempsey et al., 2019; Dixon and Hughes, 2019; Dougherty et al., 2019; Ebrahimi-Ghiri et al., 2019; Elhady et al., 2019; Estrada-Camarena et al., 2019; Faure et al., 2019; Fisch et al., 2019; Freels et al., 2019; Garbarino et al., 2019; García-Díaz et al., 2019; García-Ríos et al., 2019; Gubert and Hannan, 2019; Hatcher et al., 2019; Herbst et al., 2019; Hetzler et al., 2019; Jalilzad et al., 2019; Kosel et al., 2019; Kruse et al., 2019; Kumar et al., 2019; Laureano-Melo et al., 2019; Lee et al., 2019; Lin T. et al., 2019; Lin Y. et al., 2019; Liu et al., 2019; Lopes Andrade et al., 2019; Lovelock and Deak, 2019; Malikowska-Racia et al., 2019; Marks et al., 2019; Matsuo et al., 2019; Medawar et al., 2019; Miao et al., 2019; Miguel et al., 2019; Moreira et al., 2019; Morgan et al., 2019; Munshi et al., 2019; Nakazawa et al., 2019; Neuwirth et al., 2019b; Peleh et al., 2019; Peng et al., 2019; Queiroz et al., 2019; Samad et al., 2019; Sapozhnikova et al., 2019; Scholl et al., 2019; Suleymanova et al., 2019; Tartaglione et al., 2019; Tillmann and Wegener, 2019; Tillmann et al., 2019; Trofimiuk et al., 2019; Tsatsakis et al., 2019; Victoriano et al., 2019; Vieira et al., 2019; Wąsik et al., 2019; Wang G. et al., 2019; Wang L. et al., 2019; Wang H. et al., 2019; Winther et al., 2019; Xiao et al., 2019; Yang et al., 2019; Yuan et al., 2019; Zaccarelli-Magalhães et al., 2019; Zare et al., 2019; Zoeram et al., 2019; Bond et al., 2020; Ng et al., 2020) in which a full PDF was freely accessible were downloaded and their methods section were examined for clear reporting of the ethological lighting used (i.e., ~ ranging from 300 to 1,000 Lux to ensure to induce an anxiogenic response to light as an aversive stimulus for all tests but the Three Chamber Social Interaction Test). The Three Chamber Social Interaction Test requires a low-light stimulus in order to promote social behaviors in approaching other rodents as an anxiolytic stimulus (i.e., ~ ranging from 0 to 30 Lux to prevent freezing and other immobility behaviors that would otherwise interfere with testing). Data were included if the publication reported lighting measures, whereas if the publication did not, it was excluded (i.e., this also excluded any other work that previously cited other work). The number of remaining articles that met the criteria for these animal behavioral testing purposes were: the Light/Dark Box Test [*n* = 98 (Banaskowski et al., 2015; Bentea et al., 2015; Bertolus et al., 2015; Brown et al., 2015; Chao et al., 2015; Colla et al., 2015; Kalouda and Pitsikas, 2015; Lee K. M. et al., 2015; Lee et al., 2017; Liu et al., 2015; Livingston-Thomas et al., 2015; Pereda et al., 2015; Quines et al., 2015; Saitoh et al., 2015; Sauce et al., 2015; Skupio et al., 2015; Thompson et al., 2015; Tothova et al., 2015; Yu et al., 2015; Zhan, 2015; Zhang C. et al., 2015; Zhang et al., 2016; Acevedo et al., 2016; Christensen et al., 2016; Daniel and Hughes, 2016; Diaz et al., 2016; Farajdokht et al., 2016; Fedotova et al., 2016; Fernandez et al., 2016; Hicks J. A. et al., 2016; Hughes and Hancock, 2016; Labots et al., 2016; Li J. et al., 2016; Miranda-Morales and Pautassi, 2016; Mlyniec et al., 2016; Rojas et al., 2016; Salari et al., 2016; Scheinert et al., 2016; Socala and Wlaz, 2016; Stohn et al., 2016; Vogt et al., 2016; Abdellatif et al., 2017; Alkhlaif et al., 2017; Bahi, 2017b; Benoit et al., 2017; Borbely et al., 2017; Chandra Sekhar et al., 2017; Djordjevic et al., 2017; Domonkos et al., 2017; Khalil and Fendt, 2017; Makinson et al., 2017; Orfanidou et al., 2017; Rogers et al., 2017; Salari and Amani, 2017; Sanna et al., 2017; Scheich et al., 2017; Shafia et al., 2017; Sirohi et al., 2017; Toma et al., 2017; Wen et al., 2017; Alves et al., 2018; Amodeo et al., 2018; Cazuza et al., 2018; Dong et al., 2018; Ferreira de Araújo et al., 2018; Garcia-Gutierrez et al., 2018; Heinla et al., 2018; Hughes and Hamilton, 2018; Jacobskind et al., 2018; Keenan et al., 2018; Kosari-Nasab et al., 2018; Mahmoudi et al., 2018; Morley-Fletcher et al., 2018; Van Camp et al., 2018; Varghese et al., 2018; Walia et al., 2018, 2019a, b; Wille-Bille et al., 2018; Zhang and Yao, 2018; Zhou et al., 2018; Al-Harrasi et al., 2019; Bahi and Dreyer, 2019; Borrow et al., 2019; Dempsey et al., 2019; Dixon and Hughes, 2019; Freels et al., 2019; Laureano-Melo et al., 2019; Lovelock and Deak, 2019; Matsuo et al., 2019; Medawar et al., 2019; Morgan et al., 2019; Peng et al., 2019; Queiroz et al., 2019; Samad et al., 2019; Tillmann et al., 2019; Wang H. et al., 2019; Winther et al., 2019; Zaccarelli-Magalhães et al., 2019], the Open Field Test [*n* = 105 (Allah Yar et al., 2015; Amos-Kroohs et al., 2015; Colla et al., 2015; Dutra-Tavares et al., 2015; Fowler and Muma, 2015; Gray and Hughes, 2015; Haleem et al., 2015; Iqbal et al., 2015; Kalouda and Pitsikas, 2015; Kawasaki et al., 2015, 2017; Ketcha Wanda et al., 2015; Listowska et al., 2015; Liu et al., 2015, 2019; Nakamura A. et al., 2015; Nunes et al., 2015; Rilett et al., 2015; Thompson et al., 2015; Wensheng et al., 2015; Zagorácz et al., 2015; Biagioni et al., 2016; Dagan et al., 2016; Dos Anjos et al., 2016; Estork et al., 2016; Figueiredo et al., 2016; Girard et al., 2016; Gomez et al., 2016; Hicks K. et al., 2016; Horsley et al., 2016; Lecorps et al., 2016; Li K. et al., 2016; Martins-Júnior et al., 2016; Miller et al., 2016; Onaolapo et al., 2016; Psyrdellis et al., 2016a, b; Saré et al., 2016; Schambra et al., 2016; Tran and Keele, 2016; Wscieklica et al., 2016; Bahi, 2017b; Blankenship et al., 2017; Casarrubea et al., 2017; Donatti et al., 2017; Hansen et al., 2017; Kawabe, 2017; Khalil and Fendt, 2017; Kuniishi et al., 2017; Machado et al., 2017; Mazur et al., 2017; Rangel-Barajas et al., 2017; Reinhart et al., 2017; Sanguedo et al., 2017; Santangelo et al., 2017; Santos et al., 2017; Speight et al., 2017; Sprowles et al., 2017; Zimcikova et al., 2017; van Den Boom et al., 2017; Batinić et al., 2018; Blume et al., 2018; Bodden et al., 2018; Cui et al., 2018; Dastamooz et al., 2018; Evans et al., 2018; Holubová et al., 2018; Khalil et al., 2018; Melo-Thomas et al., 2018; Noworyta-Sokołowska et al., 2018; Perea-Rodriguez et al., 2018; Purvis et al., 2018; Rauhut and Curran-Rauhut, 2018; Robinson et al., 2018; Rojas-Carvajal et al., 2018; Roohi-Azizi et al., 2018; Saitoh et al., 2018; Struntz and Siegel, 2018; Wang et al., 2018a; Wang G. et al., 2019; Bonuti and Morato, 2018; Caliskan et al., 2019; Dougherty et al., 2019; Elhady et al., 2019; Fisch et al., 2019; Hetzler et al., 2019; Jalilzad et al., 2019; Kruse et al., 2019; Kumar et al., 2019; Lopes Andrade et al., 2019; Marks et al., 2019; Miguel et al., 2019; Neuwirth et al., 2019b; Sapozhnikova et al., 2019; Suleymanova et al., 2019; Trofimiuk et al., 2019; Tsatsakis et al., 2019; Yuan et al., 2019; Zare et al., 2019)], the Elevated Plus Maze [*n* = 99 (Kochenborger et al., 2014; Casarrubea et al., 2015, 2016; Colla et al., 2015; Daher and Mattioli, 2015; Gamberini et al., 2015; Goes et al., 2015; Hill et al., 2015; Horii and Kawaguchi, 2015; Ketcha Wanda et al., 2015; Lawther et al., 2015; Lecorps and Féron, 2015; Mascarenhas et al., 2015; Mcneilly et al., 2015; Noguerón-Merino et al., 2015; Rafati et al., 2015; Reimer et al., 2015; Słupski and Rutkowska, 2015; Saito and Brandão, 2015; Telonis and Margarity, 2015; Yang et al., 2015; Zhou et al., 2015; Bashiri et al., 2016; Cipriano et al., 2016; Dhediya et al., 2016; Duarte et al., 2016; Estork et al., 2016; Gomes et al., 2016; Kang et al., 2016; Komaki et al., 2016; Lamontagne et al., 2016; Lecorps et al., 2016; Li K. et al., 2016; Näslund et al., 2016; O’Connor et al., 2016; Palotai and Telegdy, 2016; Rico et al., 2016; Rodrigues Tavares et al., 2016; Serafim et al., 2016; Yeung et al., 2016; Akbar et al., 2017; Bartolomé et al., 2017; Bassi et al., 2017; Demir Özkay et al., 2017; Donatti et al., 2017; Fernandes et al., 2017a, b; Gatica et al., 2017; Gillette et al., 2017; Jiménez-Ferrer et al., 2017; Kędzierska et al., 2017; Kyriakou et al., 2017; Leković et al., 2017; Malikowska and Sałat, 2017; Mazur et al., 2017; Narasingam et al., 2017; Schindler et al., 2017; Taherichadorneshin et al., 2017; Vázquez-León et al., 2017, 2018; Bialuk et al., 2018; Boyette-Davis et al., 2018; Cazuza et al., 2018; Donaire et al., 2018; Funck et al., 2018; Hirano et al., 2018; López Rivilli et al., 2018; Macedo et al., 2018; Melo et al., 2018; Morud et al., 2018; Nie et al., 2018; Shimizu et al., 2018; Sorregotti et al., 2018; Sparling et al., 2018; Tavares et al., 2018; Upadhyay et al., 2018; Walia et al., 2018, 2019a; Wang S. et al., 2018; Arnold et al., 2019; Atigari et al., 2019; Caliskan et al., 2019; Ebrahimi-Ghiri et al., 2019; Estrada-Camarena et al., 2019; García-Ríos et al., 2019; Hatcher et al., 2019; Herbst et al., 2019; Kumar et al., 2019; Lee et al., 2019; Malikowska-Racia et al., 2019; Moreira et al., 2019; Munshi et al., 2019; Neuwirth et al., 2019b; Scholl et al., 2019; Tillmann and Wegener, 2019; Victoriano et al., 2019; Vieira et al., 2019; Wąsik et al., 2019; Zare et al., 2019)], and the Three Chamber Social Interaction Test [*n* = 49 (Bernard et al., 2015; Bruining et al., 2015; Cox et al., 2015; Finlay et al., 2015; Kigar et al., 2015; Kumar et al., 2015; Langley et al., 2015; Lee J. et al., 2015; Lee et al., 2016, 2018; Buffington et al., 2016; Ferri et al., 2016; Hegde et al., 2016; Kerr et al., 2016; Kratsman et al., 2016; Kumar and Sharma, 2016a, b; Lawson et al., 2016; Lee and Green, 2016; Li J. et al., 2016; de La Tremblaye et al., 2016; Bagosi et al., 2017a, b; Burke and Trang, 2017; Cai et al., 2017; Cao et al., 2017; Ebihara et al., 2017; Garcia et al., 2017; Henbid et al., 2017; Hsieh et al., 2017; Karkaba et al., 2017; Arakawa, 2018; Bausch et al., 2018; Benekareddy et al., 2018; Borland et al., 2018; Crestani et al., 2018; Goñi-Balentziaga et al., 2018; He et al., 2018; Holman et al., 2018; Leung et al., 2018; Basaure et al., 2019; Burns et al., 2019; Faure et al., 2019; Garbarino et al., 2019; García-Díaz et al., 2019; Gubert and Hannan, 2019; Kosel et al., 2019; Lin T. et al., 2019; Lin Y. et al., 2019; Bond et al., 2020)]. The following data indicate the included number of publications with adequate reporting of lighting from the starting *n* = 105 per behavioral test: the Light/Dark Box Test [*n* = 61; 62.25% (Banaskowski et al., 2015; Bentea et al., 2015; Bertolus et al., 2015; Kalouda and Pitsikas, 2015; Liu et al., 2015; Quines et al., 2015; Sauce et al., 2015; Skupio et al., 2015; Thompson et al., 2015; Yu et al., 2015; Zhang C. et al., 2015; Zhang et al., 2016; Acevedo et al., 2016; Christensen et al., 2016; Diaz et al., 2016; Farajdokht et al., 2016; Fedotova et al., 2016; Fernandez et al., 2016; Hicks J. A. et al., 2016; Labots et al., 2016; Miranda-Morales and Pautassi, 2016; Salari et al., 2016; Socala and Wlaz, 2016; Vogt et al., 2016; Bahi, 2017a; Benoit et al., 2017; Borbely et al., 2017; Chandra Sekhar et al., 2017; Domonkos et al., 2017; Khalil and Fendt, 2017; Lee et al., 2017; Makinson et al., 2017; Orfanidou et al., 2017; Rogers et al., 2017; Salari and Amani, 2017; Sanna et al., 2017; Sirohi et al., 2017; Amodeo et al., 2018; Cazuza et al., 2018; Dong et al., 2018; Ferreira de Araújo et al., 2018; Garcia-Gutierrez et al., 2018; Heinla et al., 2018; Keenan et al., 2018; Mahmoudi et al., 2018; Morley-Fletcher et al., 2018; Van Camp et al., 2018; Wille-Bille et al., 2018; Zhang and Yao, 2018; Al-Harrasi et al., 2019; Bahi and Dreyer, 2019; Borrow et al., 2019; Freels et al., 2019; Laureano-Melo et al., 2019; Lovelock and Deak, 2019; Matsuo et al., 2019; Morgan et al., 2019; Peng et al., 2019; Tillmann et al., 2019; Winther et al., 2019; Zaccarelli-Magalhães et al., 2019)], the Open Field Test [*n* = 40; 30.09% (Allah Yar et al., 2015; Fowler and Muma, 2015; Kalouda and Pitsikas, 2015; Liu et al., 2015; Nakamura A. et al., 2015; Rilett et al., 2015; Thompson et al., 2015; Wensheng et al., 2015; Dagan et al., 2016; Estork et al., 2016; Girard et al., 2016; Li K. et al., 2016; Psyrdellis et al., 2016a, b; Schambra et al., 2016; Wscieklica et al., 2016; Blankenship et al., 2017; Casarrubea et al., 2017; Hansen et al., 2017; Kawabe, 2017; Kawasaki et al., 2017; Khalil and Fendt, 2017; Kuniishi et al., 2017; Mazur et al., 2017; Speight et al., 2017; Batinić et al., 2018; Blume et al., 2018; Bodden et al., 2018; Holubová et al., 2018; Noworyta-Sokołowska et al., 2018; Perea-Rodriguez et al., 2018; Rauhut and Curran-Rauhut, 2018; Robinson et al., 2018; Rojas-Carvajal et al., 2018; Saitoh et al., 2018; Struntz and Siegel, 2018; Hetzler et al., 2019; Jalilzad et al., 2019; Kumar et al., 2019; Neuwirth et al., 2019b)], the Elevated Plus Maze [*n* = 41; 41.41% (Kochenborger et al., 2014; Casarrubea et al., 2015, 2016; Daher and Mattioli, 2015; Horii and Kawaguchi, 2015; Saito and Brandão, 2015; Yang et al., 2015; Zhou et al., 2015; Cipriano et al., 2016; Estork et al., 2016; Li K. et al., 2016; Näslund et al., 2016; Serafim et al., 2016; Akbar et al., 2017; Bartolomé et al., 2017; Fernandes et al., 2017a, b; Kyriakou et al., 2017; Mazur et al., 2017; Vázquez-León et al., 2017, 2018; Bialuk et al., 2018; Boyette-Davis et al., 2018; Cazuza et al., 2018; Funck et al., 2018; Hirano et al., 2018; López Rivilli et al., 2018; Shimizu et al., 2018; Sorregotti et al., 2018; Tavares et al., 2018; Wang S. et al., 2018; Caliskan et al., 2019; Ebrahimi-Ghiri et al., 2019; García-Ríos et al., 2019; Lee et al., 2019; Moreira et al., 2019; Munshi et al., 2019; Scholl et al., 2019; Victoriano et al., 2019; Wąsik et al., 2019)], and the Three Chamber Social Interaction Test [*n* = 17; 34.69% (Kumar et al., 2015; Langley et al., 2015; Lee J. et al., 2015; Nakamura K. et al., 2015; Ferri et al., 2016; Kerr et al., 2016; de La Tremblaye et al., 2016; Bagosi et al., 2017a, b; Cai et al., 2017; Mihara et al., 2017; Benekareddy et al., 2018; Crestani et al., 2018; He et al., 2018; Namvarpour et al., 2018; Faure et al., 2019; Nakazawa et al., 2019)]. The Zero Maze and the Light/Dark Test were excluded from the refined analyses as the Zero Maze showed very little use in the field compared to the Elevated Plus Maze and the Light/Dark Test showed equivalent use in the field compared to the Light/Dark Box Test. Figure 1 illustrates a flow chart diagram of the refined search and article sample selection method.

### Publications Meeting Criteria for Ethologically Controlled Lighting

Lastly, from the *n* = 351 selected publications, using the reporting of lighting as the next inclusion criteria, resulted in *n* = 159 publications returned across the behavioral tests (see Figure 1 middle light gray rectangles). Next, a new refinement criteria of whether the publications clearly noted the use of Lux for the evaluation of ethologically controlled lighting were determined. From this new refinement criteria (see Figure 1 lower gray rectangles) the following data indicate the included number of publications from the previously selected *n* = 159: the Light/Dark Box Test [*n* = 29 (Banaskowski et al., 2015; Bentea et al., 2015; Liu et al., 2015; Sauce et al., 2015; Zhang C. et al., 2015; Zhang et al., 2016; Acevedo et al., 2016; Christensen et al., 2016; Farajdokht et al., 2016; Fernandez et al., 2016; Hicks J. A. et al., 2016; Labots et al., 2016; Miranda-Morales and Pautassi, 2016; Vogt et al., 2016; Benoit et al., 2017; Borbely et al., 2017; Chandra Sekhar et al., 2017; Khalil and Fendt, 2017; Makinson et al., 2017; Rogers et al., 2017; Sirohi et al., 2017; Heinla et al., 2018; Keenan et al., 2018; Mahmoudi et al., 2018; Wille-Bille et al., 2018; Zhang and Yao, 2018; Laureano-Melo et al., 2019; Morgan et al., 2019; Tillmann et al., 2019)], the Open Field Test [*n* = 10 (Zhang C. et al., 2015; Estork et al., 2016; Casarrubea et al., 2017; Kawabe, 2017; Khalil and Fendt, 2017; Kuniishi et al., 2017; Blume et al., 2018; Perea-Rodriguez et al., 2018; Struntz and Siegel, 2018; Hetzler et al., 2019)], the Elevated Plus Maze [*n* = 4 (Zhang C. et al., 2015; Estork et al., 2016; Wang S. et al., 2018; Moreira et al., 2019)], and the Three Chamber Social Interaction Test [*n* = 9 (Langley et al., 2015; Lee J. et al., 2015; Nakamura K. et al., 2015; Ferri et al., 2016; Kerr et al., 2016; Mihara et al., 2017; Benekareddy et al., 2018; Crestani et al., 2018; He et al., 2018)]. Thus, from the original *N* = 420 publications sampled, only *n* = 50 met the criteria for ethologically controlled lighting across the behavioral tests evaluated herein, representing 11.9% of the sample studied (see Figure 1 white lowest rectangle).

### Statistical Analyses

**Figure 2:**
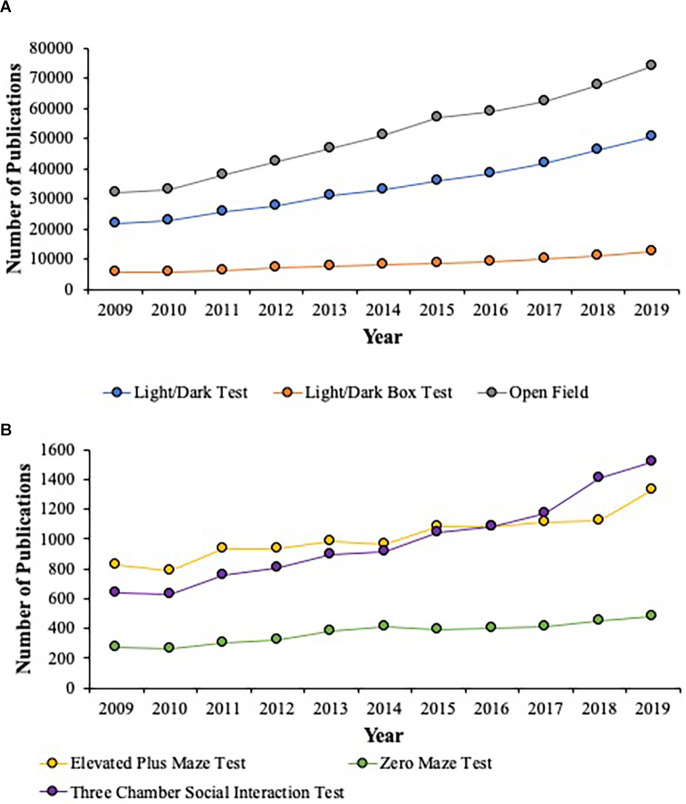
This figure illustrates the number of publications for the Light/Dark Test, Light/Dark Box Test, and the Open Field Test **(A)** and the Elevated Plus Maze, Zero Maze Test, and the Three Chamber Social Interaction Test **(B)** from 2009 to 2019 that populated from Elsevier’s Science Direct search engine. The data show that from 2009 to 2019, across all the behavioral tests noted above, there is a range of 43%–62% increase in their use across the last decade. The most popular behavioral tests used are the Open Field (Gray; **A**), the Light/Dark Test (Blue; **A**), followed by the Three Chamber Social Interaction Test (Purple; **B**), and the Elevated Plus Maze (Yellow; **B**). The Light/Dark Box Test (Orange; **A**) and the Zero Maze Test (Green; **B**) are used less than the Light/Dark Test (Blue; **A**) and the Elevated Plus Maze (Yellow; **B**). Across all behavioral tests, the Zero Maze Test was utilized the least (Green; **B**).

**Figure 3:**
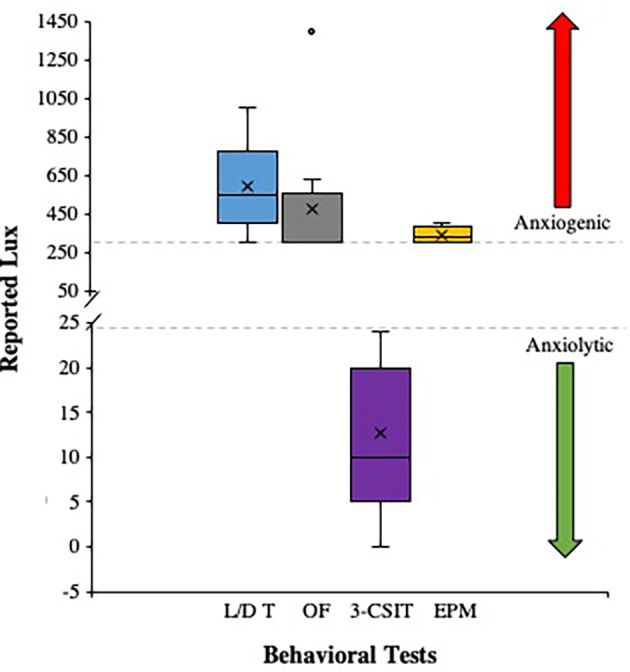
This figure illustrates the number of publications that met the criteria for ethologically relevant use of light stimuli (Lux) reported for the Light/Dark Test (L/D T), Open Field Test (OF), the Three Chamber Social Interaction Test (3-CSIT), and the Elevated Plus Maze Test (EPM). The data are presented as Box and Whisker Plots where the mean is represented as (X) the median represented as the line within the boxes (-), the *inter-quartile ranges* (*IQRs*) are represented as the lower portion of the whisker to the box (*IQR 1*), the lower box to the median (*IQR 2*), the median to the upper box (*IQR 3*), and the upper box to the upper whisker (*IQR 4*). The figure also shows a gray dashed line at 300 Lux indicating a threshold for anxiogenic behaviors that occur with light stimuli above this value (red arrow), whereas anxiolytic behaviors occur with light stimuli below the 25 Lux gray dashed line (green arrow). There was one reported outlier for the OF with a Lux of 1,400 reported, otherwise the L/D T, OF, and EPM for the studies that met criteria used comparable Lux as an anxiogenic stimulus range and the 3-CSIT Lux reported were within the anxiolytic stimulus range.

The descriptive statistics for the number of articles that met the aforementioned inclusion criterion were processed using SPSS version 24 (IBM^®^, Armonk, NY, United States). The data regarding the number of publications for the Light/Dark Test, Light/Dark Box Test, and the Open Field Test (Figure 2A) and the Elevated Plus Maze, Zero Maze Test, and the Three Chamber Social Interaction Test (Figure 2B) from 2009 to 2019 that were populated from Elsevier’s Science Direct search engine are depicted in Figure 2. The refined data that met the Lux criteria were depicted as Box and Whisker Plots showing the distribution of Lux used in each test (Figure 3). The mean is represented as (X) the median represented as the line within the boxes (-), the *inter-quartile ranges* (*IQRs*) were represented as the lower portion of the whisker to the box (*IQR 1*), the lower box to the median (*IQR 2*), the median to the upper box (*IQR 3*), and the upper box to the upper whisker (*IQR 4*; Figure 3).

## Results

### Trends in the Frequency of Behavioral Tests Used in Publications Over the Last Decade

The publications that were sampled for the behavioral tests of interest over the last decade showed that a large number of publications used the Light/Dark Test (*Mean* = 34, 138.55; *SD* = 9, 489.16) and/or the Light/Dark Box Test (*Mean* = 8, 383.64; *SD* = 2, 194.19) along with the Open Field test (*Mean* = 51, 209.5; *SD* = 14, 107.1; see Figure 2A). Additionally, the use of these behavioral tests increased from 2009 to 2019 to the following levels: the Light/Dark Test (43.45%), the Light/Dark Box Test (45.37%), and the Open Field Test (43.13%). These three behavioral tests represented the most utilized in the neuroscience field. In contrast, the last decade showed a moderate number of publications that used the Elevated Plus Maze (*Mean* = 1, 013.82; *SD* = 151.85) and/or the Zero Maze (*Mean* = 372.55; *SD* = 70.54) along with the Three Chamber Social Interaction Test (*Mean* = 986.91; *SD* = 291.46; see Figure 2B), which had the lowest representation. This latter point is most likely due to this behavioral test being the more recent to be introduced to and adopted within the behavioral neuroscience field. Moreover, the use of these behavioral tests increased from 2009 to 2019 to the following levels: the Elevated Plus Maze (62.29%), the Zero Maze (56.73%), and the Three Chamber Social Interaction Test (42.07%).

### Ethologically Relevant Findings From the Included Publications Reported Lux

The *n* = 50 publications that were included in the final analyses were compiled into a box and whisker plot to depict the distribution of Lux ranges reported for the Light/Dark Test, the Open Field, the Elevated Plus Maze, and the Three Chamber Social Interaction Tests, respectively. This was done to illustrate how researchers in the field are setting the lighting floor and ceiling threshold parameters for establishing operation for their behavioral tests, and when summarized in this illustrative way, can help facilitate the assessment of anxiogenic vs. anxiolytic stimuli parameters being properly used in the field under ethological motivational principles. The data showed a consist and ethologically relevant anxiogenic light stimuli (i.e., lighting was in the proper range to motivate the animal to respond appropriately) for the Light/Dark Test (*Mean Lux* = 593.41; *Median* = 550; *SD* = 245.82), the Open Field (*Mean Lux* = 476.5; *Median* = 300; *SD* = 345.32), the Elevated Plus Maze (*Mean Lux* = 337.5; *Median* = 325; *SD* = 47.87), and the Three Chamber Social Interaction Test (*Mean Lux* = 12.67; *Median* = 10; *SD* = 8.53; see Figure 3).

## Discussion

The results from reviewing the literature on the proper use of lighting controls for ethological motivation in behavioral neuroscience testing revealed that as per this subsample, the majority of the publications did not report Lux or evidence of proper controls for lighting over the last half-decade. This highlights some rather serious concerns for the field, as current researchers using behavioral testing techniques, and prospective training of the next generation of behavioral neuroscientists will need to address this matter head-on. A main tenant in any science is the use of proper controls, minimizing threats to internal validity, and certainly having the foresight to limit and whenever possible, attempt to eliminate extraneous variables. In doing so, the behavioral work conducted will have an increased probability of external, face, construct, and predictive validity. This latter point prevents the unnecessary use of duplication of work, addressing ethical concerns with reducing the number of rodents required for testing research hypotheses, and ultimately serves to advance science in a more efficient and meaningful way; especially, in the behavioral neuroscience field (Russell and Burch, 1959; National Research Council of The National Academies, 2003; Committee for the Update of the Guide for the Care and Use of Laboratory Animals, Institute for Laboratory Animal Research, Division on Earth and Life Studies, & National Research Council, 2010; Cardon et al., 2012; Office of Laboratory Animal Welfare, 2015). It is important to state that a limitation of the research conducted herein is that the results are limited to an editorial group sampled from one type of journal database and it is possible that the same findings may occur when sampled from other journal databases, but this remains to be tested. Notably, the findings from this study indicate that 11.9% of the sampled papers reported proper use of the Lux in their behavioral testing and this would allow other researchers to evaluate the quality of ethological motivational principles within their tests for determining how it would influence their work. The remaining 88.1% of the papers either failed to mention lighting stimuli or used incorrect lighting measures (i.e., anxiogenic light stimuli in an anxiolytic test or anxiolytic light stimuli in an anxiogenic test). The consequences of not mentioning light conditions or using incorrect lighting are quite different and the proportion of each case remains unknown. Thus, authors are strongly encouraged to measure the lighting for each behavioral test they conduct on anxiety-like behaviors to address this issue. Further, knowing the lighting conditions and then considering the anxiety-like behaviors will serve to aid authors in confirming or disconfirming whether they have observed the animals’ responses to be consistent with what is expected for a given behavioral test when conducted within the appropriate Lux range consistent with prior reports.

From the publications that met the criterion (Figure 3), there seems to be a consistent range of Lux being used for the behavioral tests as follows: the Light/Dark Box Test (300–1,000 Lux), the Open Field Test (300–635 Lux), the Elevated Plus Maze (300–400 Lux), and the Three Chamber Social Interaction Test (0–24 Lux), respectively. In particular, the anxiogenic behavioral tests (i.e., the Light/Dark Box Test, the Open Field Test, and the Elevated Plus Maze) were shown to have publications reporting Lux within overlapping ranges (Garcia et al., 2005, 2011; Miller et al., 2021; Shoji and Miyakawa, 2021). This is a good sign that some researchers are conscientious of the ethological motivational factors and by using the same and/or approximate ranges of Lux that overlap, it permits the ability to have an external, face, construct, and predicative validity for these tests. In addition, the anxiolytic Three Chamber Social Interaction Test showed a broad low range of Lux to promote mobility as it is a key principle and motivating factor to ensure rodents are comfortable and will attempt to engage in movement related to the social operationally defined dependent measures for the respective test. Moreover, what can be extracted from this study is that when researchers use these behavioral tests, they should first determine their lighting stimuli in their respective behavioral testing rooms. If need be, a commercial Lux meter can be easily purchased from the internet (e.g., for $20-$25 USD from www.amazon.com). Thus, there is no cost-prohibitive factor in securing a simple, yet critical, piece of equipment for determining Lux prior to starting a behavioral research study.

However, even though the articles that met the inclusion criteria for each test show logical use of Lux for conducting each of the behavioral tests reviewed, there are still some concerns in variability that arise and should caution how future work should be reported and reviewed carefully. For example, the consistent reporting of species used and sex studied are paramount in understanding anxiety-like behaviors both within and between species as well as sex within a given species. In further review of the articles that met the inclusion criteria (see Table 1) illustrates the following variability in sex reporting that was found: Elevated Plus Maze (25% for males, 25% for females, and 50% for both males and females); Three Chamber Social Interaction Test (50% for males, 25% for both males and females, 12.5% for females, and 12.5% did not specify any sex); Open Field Test (60% for males and 40% for both males and females); and Light/Dark Test (62.07% for males, 10.34% for females, and 24.14% for both males and females, and 3.45% did not specify any sex). Moreover, the variability in species reporting that was found indicated a range of rats, mice, voles, and gerbils. To this end, there is also a need to be cautious of the diurnal/nocturnal biological rhythms, the age, generation, and whether or not the animals were subjected to drug compounds or other genetic manipulation prior to anxiety-like behavioral testing. These factors are equally important to consider whilst ensuring that any extrapolations from testing these particular animal species and manipulated models should include a clear and consistent practice of reporting the sex, testing both sexes, and noting the Lux used.

**Table 1:** Variations in behavioral tests that met criteria for the Lux reported to motivate anxiety-like behaviors.

Behavioral test	Animal model	Sex	Lux reported
Elevated Plus Maze Test (EPM)	Long Evans rats (Neuwirth et al., 2019b)	M and F	300
			
	Balb/c mice (Estork et al., 2016)	M	400
			
	Mongolian gerbils and Sprague Dawley rats (Wang S. et al., 2018)	M	350
			
	Balb/c and Swiss Webster mice (Moreira et al., 2019)	M and F	250–300
			
Three Chamber Social InteractionTest (3-CSIT)	Mandarin vole (Wang L. et al., 2019)	F	20
			
	Swiss Webster mice (Crestani et al., 2018)	M	20
			
	Emx.1Cremice & Sprague Dawley rats (Benekareddy et al., 2018)	N/S	24
			
	C57Bl/6J mice and A/J mice (Mihara et al., 2017)	M	3–5
			
	C57Bl/6J mice (Ferri et al., 2016)	M	5
			
	Sprague Dawley rats (Kerr et al., 2016)	M and F	0
			
	C57Bl/6J mice and BTBR T + Itpr3tf/J mice (Langley et al., 2015)	M and F	20
			
	C57Bl/6J mice, BTBR T + Itpr3tf/J mice, 129Sl/SvlmJ mice (Zhang W. et al., 2015)	M	16
			
	+PA-deficient mice and WT mice (Nakamura K. et al., 2015)	M	10
			
Open Field Test (OF)	Balb/c mice (Estork et al., 2016)	M	300
			
	Sprague Dawley rats (Kuniishi et al., 2017)	M	300
			
	C57Bl/6J mice (Khalil and Fendt, 2017)	M and F	300
			
	Wistar rats (Casarrubea et al., 2017)	M	300
			
	Wistar rats (Kawabe, 2017)	M and F	300
			
	Sprague Dawley rats (Blume et al., 2018)	M and F	250–300
			
	Sprague Dawley rats (Robinson et al., 2018)	M	530
			
	California mice (Perea-Rodriguez et al., 2018)	M	1,400
			
	Long Evans rats (Hetzler et al., 2019)	M	635
			
	Long Evans rats (Neuwirth et al., 2019b)	M and F	300
			
Light/Dark Test (L/D)	CD-1 mice (Banaskowski et al., 2015)	M	900
			
	ICR mice (Liu et al., 2015)	M	300
			
	C57Bl/6J mice (Zhang C. et al., 2015)	M	600
			
	L1 heterozyhous (L1–/+) mice and WT (L1 +/+) mice (Sauce et al., 2015)	F	300
			
	XCT−/– mice and xCT +/+ mice with a C57BL/6J genetic background (Bentea et al., 2015)	M	700
			
	Sprague Dawley rats (Christensen et al., 2016)	F	1,000
			
	B6 mice (Hicks J. A. et al., 2016)	M	700
			
	ICR mice (Zhang et al., 2016)	M and F	500
	C57BL/6 N mice (Vogt et al., 2016)	M	600
			
	Wistar rats and Sprague Dawley rats (Fernandez et al., 2016)	M	400
			
	C57BL/6JOIaHsd mice& C57BL/6NCrl mice (Labots et al., 2016)	M	650
	Wistar rats (Acevedo et al., 2016)	M and F	400
			
	Wistar rats (Miranda-Morales and Pautassi, 2016)	M and F	400
			
	Wistar rats (Farajdokht et al., 2016)	M	1,000
			
	B6.129S6-Hcrttm1Ywa Orexin-deficient mice (Khalil and Fendt, 2017)	M and F	310
			
	SP/NKA, HK1, or the NK1 receptor gene-deleted (Tacr1−/–), Tac1 and Tac4 gene-deficient (Tac1−/– and Tac4−/–) mice, and C57 BL/6 mice (Borbely et al., 2017)	M	800
			
	Swiss Webster mice (Benoit et al., 2017)	M	1,000
			
	CD-1 out-bred mice (Makinson et al., 2017)	M and F	300
			
	Balb/c mice (Chandra Sekhar et al., 2017)	M	500
			
	C57BL/6J mice (Rogers et al., 2017)	N/S	700
			
	Long Evans rats (Sirohi et al., 2017)	M	600
			
	Wistar rats (Mahmoudi et al., 2018)	M	1,000
			
	C57/BL/6Arc mice, SJL mice, Swiss Webster mice, SJL/BL6 mice, C%&BL/6 mice, CD-1 mice, and Sv129 mice (Keenan et al., 2018)	M	934
			
	C57BL/6NHsd mice, and Balb/vOlaHsd mice (Heinla et al., 2018)	F	550
			
	Wistar rats (Wille-Bille et al., 2018)	M and F	400
			
	ICR mice (Zhang and Yao, 2018)	M	400
			
	Swiss Webster mice (Laureano-Melo et al., 2019)	M and F	400
			
	ICR mice (Morgan et al., 2019)	M	350
			
	Wistar rats (Tillmann et al., 2019)	M	350
			

*Note: N/S, not stated in article*.

Another factor for consideration is the issue of testing time (i.e., duration) of the anxiety-like behaviors in these species and sex used as pre-clinical models. In a review of the articles that met the inclusion criteria, it was also observed that variability ranging from as low as 4 min to as high as 30 min was used, but on average many studies reported use of 5 min to 10 min across all behavioral tests evaluated. Another concern arises that in the case of most animals, novelty to a new environment may induce natural neophobic traits that would cause the anxiogenic effect of just being exposed to a new testing environment. Thus, when constraining a behavioral test to provide an index or screening of anxiety-like behaviors, these tests may be skewed towards inflating the anxiogenic response profile as they do not offer ample time for habituation to a novel environment to be assessed carefully. In contrast, when prolonging a behavioral test of anxiety-like behaviors to run longer than 10 min, the inverse problem arises whereby the test may be skewed towards inflating the anxiolytic response profile as they offer too much time to habituate and remain uninterested in the novel environment to be assessed carefully. Thus, the 10 min behavioral testing time (i.e., duration) for a single test session for an animal is the recommended time (i.e., the first 5 min are used for screening anxiogenic responses and the last 5 min are used for screening anxiolytic responses).

Further, lighting becomes equally important when rodents are subjected to sequential behavioral tests that may compound or cause carryover anxiogenic effects [i.e., using the Open Field, then the Elevated Plus Maze, then the Two-Day Hole Board Test, or Context Fear Conditioning Tests, etc. (Neuwirth et al., 2013, 2015; Neuwirth et al., 2017; Neuwirth et al., 2019a; Neuwirth, 2014; Budylin et al., 2019)] and knowing the Lux measures can help to standardize such testing procedures to limit or as best limit artificially inflating the rodent’s anxiogenic neurobehavioral profiles. This is important as behavioral pharmacology or psychopharmacology that is used for such pre-clinical testing may then show either false-positives or false-negatives since they may represent more of an exaggerated behavioral phenotype than a well-intended pre-clinical animal model for screening new anxiolytic medications. In some cases, it may actually behoove researchers to employ the Open Field using an anxiolytic Lux stimulus (i.e., 0–24 Lux consistent with the Three Chamber Social Interaction Test) to promote behavioral movement in a baseline screening effort to assess for traits of hyperactivity or hypoactivity prior to being sequentially tested in the Elevated Plus Maze (Neuwirth et al., 2019a). This thoughtful and intentional behavioral testing methodology may help to better scrutinize whether immobility, freezing, or lack of exploration in the open arms of the Elevated Plus Maze were due to hypoactivity traits that were missed in a prior Open Field Test under anxiogenic lighting, in comparison to the rodent being hyperactive in the Open Field the prior day under anxiolytic lighting. The latter would provide insight into the clear environmental stimuli (e.g., the Elevated Plus Maze) and the lighting stimuli (i.e., anxiogenic effects), creating through the proper ethological motivational controls an actual anxiogenic behavioral response specific to the combined stimuli and not stimuli that were carried over. Additionally, through standardizing the anxiogenic and anxiolytic lighting stimuli for behavioral tests, such lighting parameters can be generalized over into novel or other behavioral tests to induce the same ethological motivational factors to make the rodents elicit specific and well-controlled behavioral responses, that in turn, can be treated with anxiolytic drugs for pre-clinical testing. Thus, with proper care and consideration for standardizing Lux as a measure in all behavioral neuroscience research design and then subsequent testing, better and more rapid advancements into anxiolytic drugs in the pre-clinical stage may become a reality for next-generation behavioral neuroscientists.

## Conclusion

The field of behavioral neuroscience has had many challenges to overcome during the last few decades. One factor studied herein was the proper use of ethologically motivated Lux/lighting stimuli in the Light/Dark Test, the Open Field, the Elevated Plus Maze (i.e., for proper anxiogenic controls), and the Three Chamber Social Interaction Test (i.e., for proper anxiolytic controls). In a review of a sample set of *N* = 420 publications using these tests (i.e., *n* = 100–102 per test with approximately *n* = 20–22 sampled from each year from 2015 to 2019 prior to the pandemic), there were only *n* = 50 publications that were properly done and specified in their reports the use of Lux. This suggests that if this sample set were to be generalized back to the total publications in the literature that approximately 11%–12% of publications use proper ethologically motivated controls in their behavioral tests. This raises serious concerns for researchers in the area of behavioral neuroscience and perhaps the following suggestions may help to leverage better publishing controls for ensuring that the behavioral neuroscience community revisits this issue and sets a new standard to address this problem as the field moves forward. First, all journals that solicit and accept manuscript submissions that use behavioral testing, must be reviewed by an expert in the field with a behavioral neuroscience background. If journals are lacking such expertise in their editorial board or review board, they should solicit experts in the behavioral neuroscience area to serve to address this deficiency. Second, the journal’s editorial board should clearly identify such individuals for the public to be informed to confirm such expertise exists for the given journal. Third, in the requirements for the author’s manuscript guidelines, a subsection should include clearly stating the Lux/lighting for each behavioral test used as part of the submission requirements [i.e., this could also include the color of the light, Lux, and if using another measure (e.g., Watt), then the distance of the light from the behavioral test apparatus ought to be added along with the type of light (e.g., LED) so that a conversion to Lux can be conducted by the reader]. If the manuscript does not have this information, it should be returned to the authors to be corrected, and if it cannot be corrected, it should be rejected (i.e., this is also true if the specie, sex, age of the animals, and *n*-size used are not reported; especially, for the use of new mutant/genetically modified animals). Fourth, a re-evaluation of this report should occur in another decade to determine whether such standards have been actually approached or achieved in a direct effort to address one challenge in the behavioral neuroscience field so that other precious efforts and resources can be devoted to other challenges left unaddressed in the area. The goal should be for the field to reach 80%–85% of publications using behavioral neuroscience methods to consistently report Lux to interpret the ethologically motivated controls from each report. Additionally, researchers in the field should also seek to achieve an equal amount of reports on both male and female animal behaviors that should also reach 80%–85% of reports as the current situation shows largely a male animal model dominated literature. The latter is a critical point as the clinical literature reports greater susceptibility, vulnerability, and diagnoses of disorders in women over men. Moreover, males and females respond differently to drugs and drugs may need to be designed specifically to their differences in neurophysiological systems. Thus, the field needs to be more cognizant and intentional in increasing female animal models to be included in their pre-clinical studies. Lastly, researchers have the utmost responsibility that if they are to work in and employ behavioral neuroscience techniques and methods in the field then it is their duty to upskill and reskill themselves in fully understanding the importance of ethologically motivated controls for their behavioral tests. This would be no different for someone needing to know the basic principles of proteins to conduct proper Western blots, or reporting the degrees of freedom for any statistical test in a manuscript. So too, the Lux measure ought to be reported in all behavioral neuroscience manuscripts as they should not be an exception. In closing, researchers in this area can find invaluable information from seminal books on behavioral neuroscience testing from a range of classics to modern resources to help fill in any gaps that one might have (Green and Swets, 1966; Underwood, 1966; Gordon et al., 1968; Richelle and Lejune, 1980; Martin, 1997; Plomin et al., 2001; Harrington, 2011; Conn, 2017; Kolb and Whishaw, 2017; Commins, 2018).

## Data Availability Statement

The original contributions presented in the study are included in the article, further inquiries can be directed to the corresponding author.

## Author Contributions

MV (Open Field Test), ZH-M (Elevated Plus Maze/Zero Maze), JO (Light/Dark Test and/or Light/Dark box), and OL (Three Chamber Social Interaction Test) conducted the literature search, the sample collection method, and organized and tabulated the data from the respective publications of the behavioral tests reported herein. LN wrote the manuscript. MV, ZH-M, OL, and LN approved the final version of the manuscript to be submitted for publication. All authors contributed to the article and approved the submitted version.

## Funding

The study was supported by a SUNY Old Westbury Faculty Development Grant (FDG) awarded to LN and a Collegiate Science Technology Entry Program (CSTEP)/LSAMP stipend awarded to OL.
